# Utilidad clínica de los biomarcadores óseos: un desafío a la variabilidad

**DOI:** 10.1515/almed-2023-0041

**Published:** 2023-07-24

**Authors:** Xavier Filella, Núria Guañabens

**Affiliations:** Servicio de Bioquímica y Genética Molecular (CDB), Hospital Clinic, IDIBAPS, Hospital Clínic de Barcelona, Barcelona, España; Servicio de Reumatología, Hospital Clinic, IDIBAPS, Universitat de Barcelona, Barcelona, España

**Keywords:** biomarcadores óseos, CTX, PINP

## Abstract

Los biomarcadores óseos son un conjunto de sustancias que son liberadas a la circulación sanguínea durante el proceso de formación y/o resorción ósea y que podemos medir en sangre y orina para obtener información sobre los trastornos metabólicos del hueso. La revisión traza una perspectiva sobre los factores que influyen en la variabilidad de los biomarcadores óseos y describe los aspectos a considerar para reducirla al máximo e interpretar los resultados de manera adecuada. La variabilidad que podemos observar en la concentración de los biomarcadores óseos engloba diversos aspectos que abarcan desde su variabilidad biológica y la variabilidad de los ensayos empleados en su medida hasta la variabilidad derivada de la influencia de numerosos factores, entre los cuales el sexo, la edad, el ejercicio, su ritmo circadiano o la dieta. Todo ello se refleja en la dificultad de establecer valores de referencia precisos. El conocimiento de esta variabilidad es el primer desafío que debe afrontar su empleo en la práctica clínica. Es necesario minimizar la variabilidad de los biomarcadores óseos controlando el máximo de variables que sea posible, así como estandarizando la preparación del paciente antes de la toma de las muestras, así como su obtención y manejo.

## Introducción

Los biomarcadores óseos (Tabla 1) son un conjunto de sustancias que son liberadas a la circulación sanguínea durante el proceso de formación y/o resorción ósea y que podemos medir en sangre y orina con la finalidad de obtener información sobre las características del remodelado óseo [1, 2]. Si bien no son útiles en el diagnóstico de la osteoporosis y no ofrecen la información necesaria para sustituir la densitometría para medir la masa ósea, sí son útiles para identificar los pacientes con mayor riesgo de fractura cuando se asocian a otros factores de riesgo y especialmente para valorar precozmente la respuesta a los tratamientos antirresortivos o a un fármaco anabólico. Por otro lado, también se ha propuesto su medición en el manejo de pacientes con otras alteraciones del hueso, desde la enfermedad ósea de Paget, el hiperparatiroidismo, la osteomalacia y la osteodistrofia renal hasta las metástasis óseas.

**Tabla 1:** Descripción de los principales biomarcadores óseos.

Acrónimo	Nombre	Proceso metabólico	Fuente	Tipo de muestra	Medida	Automatización (analizador)
CTX	Telopéptido C terminal del colágeno I	Resorción	Hueso (y otros tejidos que contienen colágeno de tipo I)	Suero/Plasma (orina)	ELISA, RIA, electroquimioluminiscencia, quimioluminiscencia	Sí (Cobas, IDS-iSYS)
FAO	Fosfatasa alcalina ósea	Formación	Osteoblastos	Suero/Plasma	Electroforesis, Colorimetría, ELISA, RIA, quimioluminiscencia	Sí (Liaison, Access/UniCel DxI, IDS-iSYS
NTx	Telopéptido N-terminal del colágeno de tipo I	Resorción	Hueso (y otros tejidos que contienen colágeno de tipo I)	Orina	ELISA, RIA, quimioluminiscencia	Sí (Vitros)
OC	Osteocalcina	Formación	Osteoblastos, dentina, cartílago hipertrófico	Suero/Plasma (orina)	ELISA, RIA, electroquimioluminiscencia, quimioluminiscencia	Sí (Cobas, IDS-iSYS, mmulite, Liaison)
PINP	Propéptido N-terminal del colágeno tipo I	Formación	Hueso (y otros tejidos que contienen colágeno de tipo I)	Suero/Plasma	ELISA, RIA, quimioluminiscencia	Sí (Cobas, IDS-iSYS)
SC	Esclerostina	Inhibe formación ósea	Osteocitos, condrocitos	Suero/Plasma	ELISA, quimioluminiscencia	Sí (Liaison)
TRAP-5b	Fosfatasa ácida tartrato resistente	Resorción	Osteoclastos	Suero/Plasma	Colorimetría, RIA, ELISA, quimioluminiscencia	Sí (IDS-iSYS)
FGF-23	Factor de crecimiento fibrobástico-23	Reabsorción proximal de fosfato. Disminución de 1,25 OH vitamina D y de PTH	Oscteocitos	Plasma	ELISA, quimioluminiscencia	Sí (Liaison)

Una correcta interpretación clínica de los biomarcadores óseos debe tener en cuenta su variabilidad [3]. Este concepto engloba desde la variabilidad analítica de los ensayos empleados en su medida hasta la variabilidad preanalítica derivada de la influencia de numerosos factores, entre los cuales se incluyen el sexo, la edad, el ritmo circadiano, el ejercicio físico, una fractura reciente o la dieta. Asimismo, la falta de estandarización de los ensayos comerciales utilizados en la medida de los biomarcadores óseos dificulta su aplicación clínica. El conocimiento de los factores que motivan esta variabilidad es el primer desafío que debe afrontar su empleo en la práctica clínica.

Un documento conjunto firmado en 2010 [4] por la International Osteoporosis Foundation (IOF) y la International Federation of Clinical Chemistry and Laboratory Medicine (IFCC) recomendó la medición en suero del propéptido N-terminal del procolágeno de tipo I (PINP) como biomarcador de formación ósea y del telopéptido isomerizado β C-terminal del colágeno de tipo I (CTX) como biomarcador de resorción. El PINP se forma, por acción de proteinasas específicas, durante el procesamiento del procolágeno de tipo I que sintetizan fibroblastos y osteoblastos a colágeno de tipo I. Por su lado, el CTX se forma en el proceso de degradación del colágeno de tipo I por acción de diversas proteasas que son liberadas por los osteoclastos durante el proceso de resorción ósea.

Ambos biomarcadores fueron seleccionados para realizar estudios clínicos en osteoporosis y se han aplicado al manejo de pacientes con osteoporosis en función de diversos criterios, que incluían tanto la disponibilidad de estudios clínicos como el conocimiento de su variabilidad biológica y analítica, el fácil manejo de las muestras, la estabilidad de los analitos, la facilidad de análisis y el hecho que su medición se realiza en suero y no en orina. En la actualidad, disponemos de ensayos que permiten medir PINP y CTX en los analizadores IDS-iSYS (Immunodiagnostic Systems) y Cobas (Roche), si bien la medición de PINP que se realiza en ambos instrumentos presenta algunas diferencias que deben ser conocidas. Existen dos formas circulantes de PINP, la intacta o trimérica y la monomérica. Mientras que el ensayo de IDS-iSYS mide el PINP intacto, el ensayo de Cobas mide tanto la forma trimérica como la monomérica (PINP total). Ello es una diferencia importante en pacientes con una nefropatía, puesto que el PINP monomérico se ve afectado por la insuficiencia renal, mientras que el PINP trimérico no está influido por cambios en la función renal. Por otro lado, el PINP intacto puede ser medido también mediante un radioinmunoensayo de Orion, mientras que el PINP total puede ser medido con un ELISA de USCN Life Science Inc.

El telopéptido N-terminal del colágeno de tipo I (NTX) se forma, al igual que CTX, en el proceso de degradación del colágeno de tipo I, si bien en esta ocasión corresponde al fragmento N-terminal. Puede ser medido en orina mediante un test de ELISA o bien por quimioluminiscencia en un analizador Vitros (Ortho Clinical Diagnostics). Para ello debe recogerse la segunda orina de la mañana, descartándose las orinas contaminadas con sangre. Los resultados de NTX en orina se expresan en relación a la concentración de creatinina medida en la muestra, por lo que en el resultado medido se suma la variabilidad analítica de ambos ensayos.

Actualmente disponemos de anticuerpos monoclonales específicos que permiten la medición tanto de la fosfatasa alcalina ósea como de la fosfatasa ácida resistente al tartrato (TRAP-5b), biomarcadores que reflejan, en el primer caso, la actividad de osteoblastos, y, en el segundo caso, de forma indirecta el número de osteoclastos. La medida de fosfatasa alcalina ósea es útil en el manejo de pacientes con enfermedad de Paget y de pacientes con osteoporosis, presentando una baja reactividad cruzada con otros isoenzimas de la fosfatasa alcalina. Por otra parte, TRAP-5b es una isoforma resistente a la inhibición con tartrato de la familia de fosfatasas ácidas y que, a diferencia de la fracción 5a que se halla presente en macrófagos, se expresa en osteoclastos.

Existen, además de test de ELISA manuales, test automatizados para medir ambos biomarcadores en el analizador IDS-iSYS, mientras que la fosfatasa alcalina ósea está automatizada también en los analizadores Liaison (Diasorin), así como en Access y Unicell DxI (Beckman Coulter). La disponibilidad de inmunoensayos para medir ambos biomarcadores ha permitido soslayar muchos de los inconvenientes de otras técnicas, como la electroforesis o la colorimetría, que fueron utilizadas hasta hace unos años en su medición.

La osteocalcina, producida por los osteoblastos, es la proteína no colágena más importante de la matriz ósea. Además de en la formación ósea, está implicada en la regulación del metabolismo energético a través de su efecto sobre las células beta-pancreáticas y los adipocitos, aportando así la energía necesaria para el proceso de remodelado óseo. Si bien durante años se utilizó profusamente como un biomarcador de formación ósea, en la actualidad ha quedado relegada a algunas indicaciones, siendo, por ejemplo, un predictor útil del riesgo de desarrollar diabetes mellitus en pacientes tratados con glucocorticoides o la monitorización de la osteoporosis inducida por glucocorticoides y la asociada a colestasis crónica [5]. En la sangre están presentes tanto la osteocalcina intacta (aminoácidos 1–49) como el fragmento medio N terminal (N-mid) (aminoácidos 1–43), considerablemente más estable. La mayoría de ensayos disponibles para medir osteocalcina, muchos de ellos automatizados en distintas plataformas (Cobas, IDS-iSYS, Immulite, Liaison), determina tanto la molécula intacta como el fragmento N-mid.

La esclerostina, producida por los osteocitos, es uno de los principales reguladores de la formación ósea, y no es propiamente un marcador del recambio óseo. Su alteración, según aumente o disminuya su función, se relaciona respectivamente con enfermedades que cursan con osteoporosis u osteoesclerosis, siendo su medida particularmente interesante en pacientes con osteoporosis severa. Recientemente se dispone de un test automatizado en el analizador Liaison que permite su medida, si bien también puede ser medida mediante test de ELISA.

El factor de crecimiento fibrobástico 23 (FGF-23) es una proteína que forma parte del grupo de las fosfatoninas y que es sintetizada por el osteocito. Actúa inhibiendo la reabsorción proximal de fosfato, a la vez que bloquea la síntesis y liberación de PTH y disminuye la concentración de 1,25 OH vitamina D al inhibir la 1 alfa hidroxilasa renal. La actividad desarrollada por FGF-23 se realiza mediante su unión al complejo formado por el receptor de FGF y klotho, proteína que actua incrementando la afinidad del receptor de FGF por FGF-23. Existen diversos ensayos de ELISA que permiten medir la forma intacta biológicamente activa de FGF-23 (entre los cuáles Immunotopics y Kainos), mientras que la fracción C-terminal puede ser medida con un ELISA de Immunotopics. Desde 2017 se dispone de un test automatizado en el analizador Liaison que mide el FGF-23 intacto y que además cuenta con marca CE.

## Variabilidad biológica

La variabilidad biológica, un concepto esencial para poder interpretar acertadamente un biomarcador, incluye la variabilidad biológica intraindividual y la variabilidad biológica interindividual. La primera responde a las fluctuaciones fisiológicas alrededor del punto de equilibrio a lo largo del tiempo de un determinado biomarcador en un mismo individuo, mientras que la segunda corresponde a la variación observada entre los distintos puntos de equilibrio de diferentes individuos.

El conocimiento de la variabilidad biológica es importante para determinar si un cambio en la medida de un biomarcador tras un tratamiento debe ser considerado como una respuesta clínicamente significativa. El valor de referencia de un cambio en la concentración de un biomarcador, también conocido como mínimo cambio significativo, deriva de la estimación de su variabilidad analítica junto a su variabilidad biológica intraindividual. Su conocimiento es de ayuda para monitorizar un tratamiento antirresortivo o anabólico y además estimar el grado de adherencia al tratamiento del paciente, objetivos que constituyen la principal indicación de los biomarcadores óseos.

Es conocida desde hace tiempo la variabilidad biológica de los biomarcadores óseos [6, 7] a causa de los múltiples factores que influyen en el remodelado óseo y que incluyen, entre otros, su regulación humoral, la dieta o la actividad física. La base de datos de variación biológica de la European Federation of Clinical Chemistry and Laboratory Medicine (EFLM) [8] recoge los resultados disponibles al respecto, si bien constata que, en general, la calidad de la mayoría de estudios publicados es baja y calificados con el grado C, según la herramienta de evaluación denominada Biological variation data critical appraisal checklist (BIVAC).

Un estudio promovido por el grupo de trabajo en variación biológica de la EFLM se ha propuesto subsanar este inconveniente. Para realizar dicho estudio se recogieron muestras de plasma EDTA en ayuno de 38 hombres y 53 mujeres a lo largo de diez semanas consecutivas, obteniéndose una muestra semanal entre las 8 y las 10 de la mañana. El estudio documenta una variabilidad biológica intraindividual de 15,1 % (IC 95 %: 14,4–16 %) para CTX, siendo menor para PINP (8,8 %; IC 95 %: 8,4–9,3 %) y para osteocalcina (8,9 %; IC 95 %: 8,5–9,4 %) [9]. Por otro lado, el grupo de trabajo conjunto de la IOF y la European Calcified Tissue Society recomienda medir CTX y PINP en el momento basal y a los tres meses del inicio del tratamiento antirresortivo, valorando como el cambio mínimo significativo una disminución de más del 56 % para CTX y del 38 % para PINP [10].

En relación a NTX, Eastell et al. [11] indican una variabilidad intraindividual a corto plazo (recogiendo muestras tres días consecutivos) del 13,1 % para NTX en orina, mientras que la variabilidad intraindividual a largo plazo (recogiendo muestras durante 2 meses tras un muestreo basal) es del 15,6 %. Según este estudio, para considerar un cambio mínimo significativo tras un tratamiento antirresortivo se requiere una disminución de NTX superior al 31 %.

Según datos del grupo de trabajo en variación biológica de la EFLM, la variabilidad biológica intraindividual del FGF-23 intacto es de 13,9 % (IC 95 %: 13,2–14,7) [9], mientras que, por otro lado, un estudio publicado por Smith et al. [12] documenta que la variabilidad intraindividual de la fracción C-terminal de FGF-23 es menor que la del FGF-23 intacto (8,3 vs. 18,3 %).

La variabilidad biológica reportada para otros biomarcadores óseos es menor. Álvarez et al. [7] indican para la fosfatasa alcalina ósea una variabilidad biológica intraindividual del 3,4 % en sujetos sanos y del 4,9 % en pacientes con enfermedad ósea de Paget. Finalmente, la base de datos de variación biológica de la EFLM indica una variabilidad biológica intraindividual del 6,6 % para la fosfatasa alcalina ósea y del 10,8 % para la fosfatasa ácida resistente al tartrato [8].

## Variabilidad analítica

Desde una perspectiva analítica debe considerarse la variabilidad del ensayo, tanto en referencia a la variabilidad intraensayo como a la variabilidad interensayo, más bajas en los ensayos automatizados que en los manuales. Por otro lado, debemos también considerar la variabilidad existente entre los diversos ensayos comerciales para medir cada uno de los biomarcadores óseos, puesto que no existe ni un método de referencia ni tampoco material de referencia para la calibración.

Un documento del Comité Conjunto sobre Metabolismo Óseo de la IOF y la IFCC para valorar la armonización de los ensayos para medir PINP subraya que los resultados son semejantes al efectuar su medición en los analizadores IDS-iSYS y Cobas cuando el filtrado glomerular es mayor a 30 mL/min/1,73 m^2^, pero que existe un sesgo significativo entre estos ensayos y el RIA de Orion [13]. También Guañabens et al. [14] observan un sesgo escaso al valorar en una población de mujeres premenopáusicas los ensayos de PINP automatizados en IDS-iSYS y Cobas. En este trabajo, en cambio, los autores comunican un mayor sesgo entre ambos equipos en la medición CTX. Esta es también la conclusión del informe del Comité Conjunto sobre Metabolismo Óseo de la IOF y la IFCC que no obstante señala que las diferencias son mayores cuando la medición se efectúa en suero, por lo que recomiendan el empleo de plasma EDTA para la medición de CTX especialmente en estudios en que hay que emplear muestras congeladas [15].

Estas diferencias complican la comparación de resultados cuando se utilizan ensayos distintos para medir una misma sustancia y dificulta tanto la adopción de valores de referencia homogéneos para la toma decisiones clínicas, como el aprovechamiento de la experiencia clínica acumulada con los diversos reactivos. El Comité Conjunto sobre Metabolismo Óseo de la IOF y la IFCC para solventar esta situación propone preparar materiales de referencia internacionales conmutables y desarrollar procedimientos de medición comunes para PINP y CTX en sangre. Ello debería permitir la definición de intervalos de referencia comunes y límites de decisión universalmente aceptables [16].

La deficiente transferibilidad de resultados entre distintos ensayos comerciales también afecta al resto de biomarcadores. Así, Schafer et al. [17] señalan diferencias en la medición de NTX entre el ensayo Osteomark y Vitros, mientras Eastell et al. [18] han comunicado una diferencia sustancial en la medición de la fosfatasa alcalina ósea mediante el test Metra BAP EIA y el test automatizado en el analizador Access. De igual manera, existen notables diferencias en los intervalos de referencia recomendados por los diversos fabricantes de ensayos para medir osteocalcina, pese a que la mayoría miden tanto la molécula intacta como el fragmento N-mid [19]. También se han documentado las discrepancias entre los diversos ensayos para medir esclerostina que cuentan también con intervalos de referencia distintos [20]. Tampoco son extrapolables las concentraciones de FGF-23 obtenidas con distintos ensayos comerciales que, además, expresan los resultados en unidades distintas [21].

## Variabilidad en la definición del intervalo de referencia de los biomarcadores óseos

La definición de un intervalo de referencia para los diversos biomarcadores óseos es fundamental para su correcta aplicación clínica. En primer lugar, porque el objetivo de la terapia antirresortiva con bifosfonatos es reducir los niveles de los biomarcadores óseos a la mitad inferior del intervalo de referencia considerado como normal para mujeres premenopáusicas. En segundo lugar, para disponer de un nivel de corte que permita valorar la capacidad de los biomarcadores óseos para predecir, junto con otros factores, el riesgo de fractura.

Sin embargo, pese a la importancia de estos objetivos, no disponemos de intervalos de referencia robustos, con diferencias en ocasiones notables pese al empleo de un mismo ensayo. La disponibilidad de ensayos estandarizados que permitan la obtención de resultados comparables con los distintos test es, por tanto, un paso indispensable para disponer de valores de referencia robustos, pero necesariamente debería ir acompañado de estudios multicéntricos que cuenten con una óptima selección de la población incluida, si bien ciertamente se han hecho esfuerzos en este sentido [14, 18, 22].

Para mujeres, el intervalo de referencia se basa en las concentraciones obtenidas en mujeres premenopausicas, con una baja tasa de recambio y con un nivel óptimo de salud ósea, mientras que en hombres se consideran las concentraciones obtenidas entre los 40 y 60 años [2].

Las diferencias en cuanto a los intervalos de referencia suelen ser mayores para CTX que para PINP. Así, Morris et al. [23] comunican que los límites inferior y superior del intervalo de referencia obtenidos en mujeres premenopáusicas con un analizador Cobas para CTX oscilan entre 70 y 163 ng/L y entre 274 y 800 ng/L, respectivamente. En cambio, son menores las diferencias indicadas para PINP, cuyo límite inferior oscila entre 14,6 y 22,7 μg/L y cuyo límite superior oscila entre 42,9 y 90 μg/L.

También Schini et al. [2] indican que, para mujeres premenopausicas, el límite inferior del intervalo de referencia para CTX oscila entre 70 y 163 ng/L, mientras que el límite superior lo hace entre 274 y 895 ng/L. En cambio, el rango diferencial para los límites inferior y superior para PINP es menor: entre 13,72 y 22,7 μg/L y entre 42,9 y 99 μg/L, respectivamente. Este mismo estudio documenta que para hombres el intervalo de referencia para CTX oscila entre 93 y 252 ng/L para el límite inferior y entre 400 y 1116 ng/L para el límite superior. En el caso de PINP, el rango diferencial para los límites inferior y superior estuvo entre 16,9 y 29,4 μg/L en el primer caso y entre 43,9 y 98 μg/L en el segundo caso.

Es significativo el trabajo publicado por Glover et al. [22] para valorar las diferencias geográficas en el intervalo de referencia de los biomarcadores óseos. En este estudio se miden PINP, CTX, NTX y fosfatasa alcalina ósea en 637 mujeres premenopáusicas sanas y con menstruaciones cíclicas regulares que viven en el Reino Unido, Francia, Bélgica y los Estados Unidos. Los autores no encuentran diferencias en las concentraciones de NTX urinario y fosfatasa alcalina ósea, pero sí para las concentraciones de PINP y CTX. Así, CTX fue significativamente más elevado en Francia en relación al Reino Unido, mientras que PIPN era más elevado en Francia y en Bélgica en relación al Reino Unido y también en Francia respecto a Estados Unidos. El trabajo también estudió distintas variables, observado diferencias significativas entre las mujeres de los diversos países en relación al índice de masa corporal, peso, embarazos previos, 25-OH vitamina D, tabaquismo, ejercicio regular y consumo de alcohol. Probablemente son estas diferencias las que se reflejan en las distintas concentraciones de PINP y CTX en las cuatro poblaciones estudiadas.

La mayoría de autores, no obstante, coincide en observar diferencias en relación a la edad y el sexo, con una concentración más elevada en hombres que en mujeres y también en individuos mayores respecto a los adultos jóvenes, siendo, obviamente su concentración mucho más elevada a lo largo de la infancia. También hay coincidencia en describir un patrón en L, con un cierto descenso en la concentración de los biomarcadores óseos entre los 30 y los 35 años y una posterior estabilización entre los 35 y los 45 años [13, 24].

Un trabajo publicado por Adami et al. [25] contribuye a entender la dificultad de establecer con precisión un intervalo de referencia para los biomarcadores óseos. Los autores miden PINP, CTX y osteocalcina en 638 mujeres premenopáusicas con edades comprendidas entre los 20 y los 50 años que clasifican en seis grupos de edad. El estudio corrobora el patrón en L de estos biomarcadores y la estabilización en mujeres con edades comprendidas entre los 35 y 50 años, pero también constata que hay un subgrupo de mujeres con una concentración de FSH superior a 30 UI/mL que, pese a tener menstruaciones cíclicas regulares, tienen una concentración de PINP, CTX y osteocalcina significativamente más elevada.

Existen pues divergencias en los intervalos de referencia documentados en los diversos estudios referidos, con rangos en ocasiones algo distintos. Pese a ello, es interesante indicar los datos comunicados por Eastell et al. [18] en un estudio que valora el intervalo de referencia de CTX, PINP, NTX y fosfatasa alcalina ósea en una población de 194 mujeres francesas y danesas premenopáusicas. Los autores destacan la semejanza en las medias obtenidas en su estudio con las de otros cuatro estudios publicados previamente.

## Factores que contribuyen a la variabilidad de los biomarcadores óseos

Son numerosas las fuentes de variabilidad que afectan la concentración de los biomarcadores óseos (Tabla 2). Su conocimiento y control puede permitir un mejor aprovechamiento de su medida. Algunos de ellos, como son el sexo y la edad, han sido ya comentados en el apartado anterior.

**Tabla 2:** Factores que contribuyen a la variabilidad de los biomarcadores óseos.

Factor	Efecto
Ritmo circadiano	Mayor concentración de los biomarcadores de resorción, en particular CTX, por la noche, con menor concentración por la tarde. La osteocalcina sigue un ritmo circadiano semejante
Ritmo estacional	En general, concentración más elevada de osteocalcina, fosfatasa alcalina ósea y NTX en invierno
Función renal	Su alteración afecta CTX, NTX y osteocalcina. La función renal también puede afectar la concentración de PINP total, pero no la de su forma intacta
Función hepática	La función hepática puede afectar la concentración de CTX, NTX y PINP
Edad y sexo	Concentración más elevada en hombres que en mujeres y también en individuos mayores respecto a los más jóvenes, siendo, obviamente su concentración mucho más elevada a lo largo de la infancia, en relación al crecimiento
Menopausia	El recambio óseo se incrementa con el inicio de la menopausia, con aumento en la concentración de los biomarcadores óseos
Ciclo menstrual	Menor resorción ósea durante la fase lútea
Embarazo	Durante el embarazo, la concentración de los biomarcadores óseos se eleva, descendiendo posteriormente tras el parto
Anticonceptivos	Influencia de los anticonceptivos en los biomarcadores óseos, en particular sobre PINP, cuya concentración disminuye
Ejercicio físico	Aumento de la resorción ósea en caso de inmovilización prolongada. El ejercicio físico intenso aumenta la formación ósea y disminuye la resorción
Ingesta de alimentos	La ingesta de alimentos en las horas previas a la toma de la muestra afecta particularmente los biomarcadores relacionados con la resorción ósea
Dieta y estilo de vida	El contenido en calcio de la dieta, la toma de suplementos de vitamina D o de calcio, el consumo de alcohol y el tabaquismo, así como el grado de sedentarismo

El ritmo circadiano [26] es el primer factor a tener en cuenta y el que va a determinar que pueda ser necesario recoger la muestra a primera hora de la mañana. El CTX tiene un sustancial ritmo circadiano, observándose que el pico máximo se observa entre las 1:30 y las 4:30 de la madrugada, mientras que el mínimo se observa entre las 11 y las 15 horas (2). Esta fluctuación puede reducirse recogiendo la muestra en ayunas y en un horario preestablecido. También se ha observado que la osteocalcina tiene un ritmo circadiano, de manera que su concentración cae a lo largo de la mañana, aumenta al anochecer, y alcanza un pico máximo durante la noche [27]. Bollen et al. [28] han descrito que el índice de NTX en relación a la creatinina en orina sigue un ritmo circadiano, de manera que cae a lo largo del día hasta un valor mínimo a última hora de la tarde. También el FGF-23 tiene, sobre todo cuando se mide la forma intacta, un importante ritmo circadiano, de manera que su concentración disminuye a lo largo de la mañana, hasta alcanzar un mínimo al mediodía y posteriormente va ascendiendo hasta alcanzar un pico máximo a medianoche [29]. Por ello, se recomienda realizar la extracción de sangre entre las 8 y las 10 de la mañana. En cambio, el ritmo circadiano de PINP y fosfatasa alcalina ósea es de menor amplitud [26].

También se han observado cambios estacionales en la concentración de los biomarcadores óseos, que estarían relacionados con el aumento de recambio óseo durante el invierno, debido, al menos en parte, a los cambios en las concentraciones de vitamina D y PTH [30]. Así, se han descrito concentraciones más elevadas de osteocalcina, fosfatasa alcalina ósea y NTX en invierno [31], sin que, sin embargo, haya unanimidad de resultados al respecto.

Por otro lado, la función hepática puede afectar la concentración de CTX, NTX y PINP, mientras que la función renal puede afectar la concentración de CTX, NTX y osteocalcina [32]. La función renal también puede afectar la concentración de PINP monomérico, pero no la de su forma intacta. La fosfatasa alcalina ósea presenta cierta reactividad cruzada con otros isoenzimas, incluyendo la hepática, por lo que podría apreciarse un ligero aumento en pacientes con hepatopatías.

Ya se han comentado las diferencias en las mujeres en función de la edad, con cambios importantes en la concentración de los biomarcadores óseos alrededor de la menopausia. También se han observado cambios en relación al ciclo menstrual a causa de la menor resorción durante la fase lútea, aunque de escasa trascendencia [33]. Asimismo, se ha descrito la influencia de los anticonceptivos en los biomarcadores óseos, en particular sobre PINP [13]. Durante el embarazo, la concentración de los biomarcadores óseos se eleva, descendiendo posteriormente tras el parto en el curso de un año.

Es también evidente la influencia del ejercicio físico en el metabolismo del hueso, conociéndose que la inmovilización prolongada causa un aumento de la resorción ósea, con el consiguiente aumento en la concentración de los biomarcadores óseos. Existen numerosos trabajos que evalúan la influencia del ejercicio físico sobre los biomarcadores óseos, valorando desde el ejercicio puntual al entrenamiento prolongado. No obstante, los resultados de los distintos trabajos no son siempre coincidentes [34].

Se han descrito cambios en relación a la ingesta que afecta particularmente los biomarcadores relacionados con la resorción ósea. La toma de alimentos reduciría la resorción ósea por un mecanismo no bien conocido, si bien algunos autores indican que podría estar mediada por la liberación de péptidos intestinales y/o pancreáticos [35]. Clowes et al. [36] recomiendan la recogida de las muestras en ayuno, si bien indican que la influencia de la ingesta de alimentos es, en general, reducida, a excepción del caso de CTX, para el cual remarcan una diferencia entre ayuno e ingesta del 17,8 %.

Por último, debemos mencionar que la dieta y el estilo de vida también pueden influir en la concentración de los biomarcadores óseos, tal como muestra el referido estudio de Glover et al. [23]. Entre los factores a mencionar se incluyen el contenido en calcio de la dieta, la toma de suplementos de vitamina D o de calcio, el consumo de alcohol, el tabaquismo y el mayor o menor sedentarismo.

En un estudio multicéntrico realizado en Cataluña en que se valora la influencia sobre las concentraciones de CTX, NTX y PINP de la edad, índice de masa corporal, tabaquismo, ingesta de calcio y de alcohol y concentración de 25 OH vitamina D y PTH, Guañabens et al. [14] comunican una mayor variabilidad para CTX (observando diferencias estadísticamente significativas en relación a edad, índice de masa corporal, ingesta de calcio y concentración de PTH) que para NTX (registrando diferencias estadísticamente significativas solo en relación a la edad) o para PINP (sin que se observaran diferencias en relación a ninguna de las variables analizadas).

Menos conocidos son los factores que afectan la concentración de FGF-23. Por un lado, se ha observado una mayor concentración de FGF-23 en individuos mayores, que podría estar relacionado con la concentración más baja de Klotho en estos sujetos. No está clara la relación de la concentración de FGF-23 con el sexo, si bien se conoce que los estrógenos disminuyen su concentración. Finalmente, hay resultados contradictorios respecto a su asociación con la etnia, mientras que sí se ha observado una concentración más elevada de FGF-23 en individuos con un nivel socioeconómico bajo, que se relacionaría con una concentración más elevada de fosfatos en estos sujetos [37].

En otro orden de cosas, debe mencionarse que algunas enfermedades (enfermedades inflamatorias, como la artritis reumatoide, hepatopatías o enfermedades que cursan con malabsorción, como la celiaquía o la enfermedad de Crohn), así como diversos tratamientos (Tabla 3) afectan la concentración de los biomarcadores óseos [2, 38, 39].

## Conclusiones

La variabilidad es el principal desafío que debemos afrontar para poder utilizar con éxito los biomarcadores óseos en la práctica clínica. Por este motivo debemos proceder a controlar el máximo de variables que sea posible (Tabla 4), procediendo a estandarizar la preparación del paciente y la obtención y el manejo de las muestras, considerando además en todos los casos la estabilidad de cada biomarcador (Tabla 5) [40].

**Tabla 3:** Tratamientos que afectan los biomarcadores óseos.

Tratamientos	Efecto en los biomarcadores óseos
Anabólicos (Teriparatida)	Aumento de los biomarcadores de formación ósea, especialmente PINP. Los marcadores de resorción aumentan más lentamente y con menor magnitud
Antirresortivos	Rápida disminución de los biomarcadores de resorción ósea, mientras que los de formación disminuyen más lentamente. Mayor disminución en tratamientos con denosumab con respecto a los bisfosfonatos
Antiandrógenos	Aumento de los biomarcadores relacionados con el remodelado
Antagonistas de la vitamina K	Ligera reducción de la osteocalcina, pero aumento de la osteocalcina no carboxilada
Anticomiciales	Discreto aumento de los biomarcadores relacionados con el remodelado
Anticonceptivos	Reducen la concentración de los biomarcadores óseos, espacialmente de formación, a destacar PINP
Glucocorticoides	Disminución de osteocalcina y PINP. Poca afectación en los biomarcadores relacionados con la resorción ósea, excepto en fases iniciales
Metotrexate	Disminuye biomarcadores relacionados con la formación, como fosfatasa alcalina y osteocalcina, mientras aumentan los relacionados con la resorción
Suplementos de calcio	Reducen la concentración de los biomarcadores óseos
Tiazidas	Discreta disminución de los biomarcadores óseos

**Tabla 4:** Recomendaciones generales para la obtención de muestras.

– Preparación del paciente antes de la toma de la muestra: evitar el ejercicio intenso
– Recoger la muestra entre las 7:30 y las 10 de la mañana, tras un ayuno de 8 horas
– Recoger segunda orina de la mañana si hay que medir NTX, que se expresará en función de la creatinina
– Considerar las condiciones de estabilidad del analito que deba ser medido: gran estabilidad de la fosfatasa alcalina ósea, mayor estabilidad de PINP que de CTX, poca estabilidad de osteocalcina
– Evitar la hemólisis o la presencia de sangre en la orina: interfiere en la medida de osteocalcina y NTX
– Tener en cuenta los cambios de ensayo en la medida de un biomarcador: realizar estudios de comparación, informar a los servicios clínicos en caso de que haya cambios

En primer lugar, deben minimizarse las fuentes preanalíticas de variabilidad. Así, se deberá conseguir una adecuada preparación del paciente antes de la toma de la muestra, de manera que evite el ejercicio intenso el día antes (Tabla 3).

**Tabla 5:** Estabilidad de los biomarcadores óseos a temperatura ambiente y a 2–8 °C.

Acrónimo	Nombre	Estabilidad a temperatura ambiente y a 2–8 °C
CTX	Telopéptido C terminal del colágeno I	A temperatura ambiente: 8 h
		Puede conservarse a 2–8 °C durante 8 h
		Mayor estabilidad en plasma EDTA: 24 h a temperatura ambiente y 8 días a 2–8 °C
FAO	Fosfatasa alcalina ósea	Puede conservarse a 2–8 °C durante 3 días
NTx	Telopéptido N-terminal del colágeno de tipo I	A temperatura ambiente: 24 h
		Puede conservarse a 2–8 °C durante 72 h
OC	Osteocalcina	A temperatura ambiente: 1 h sin separar en suero y 8 h sin separar en plasma EDTA; 8 h tras la separación
		Puede conservarse a 2–8 °C durante 72 h
PINP	Propéptido N-terminal del colágeno tipo I	A temperatura ambiente: 24 h
		Puede conservarse a 2–8 °C durante 5 días
SC	Esclerostina	La muestra debe ser centrifugada tan pronto como sea posible, preferentemente a 2–8 °C
		Las muestras son estables a temperatura ambiente hasta 4 h
		Puede conservarse a 2–8 °C durante 24 h
TRAP-5b	Fosfatasa ácida tartrato resistente	A temperatura ambiente: 8 hs
		Puede conservarse a 2–8 °C durante 3 día
FGF-23	Factor de crecimiento fibrobástico-23	La muestra puede ser mantenida a una temperatura de 2°–8 °C un máximo de 8 h tras la extracción
		Si la medición no se realiza en el plazo de 8 h la muestra debe ser congelada

En segundo lugar, deben estandarizarse las condiciones de la recogida de la muestra. La muestra, especialmente si se solicita CTX, debe ser obtenida tras un ayuno de 8 horas, entre las 7:30 y las 10 de la mañana, mientras que para medir NTX se requiere obtener la segunda orina de la mañana. También deberán tenerse en cuenta las condiciones de conservación de la muestra (gran estabilidad de la fosfatasa alcalina ósea; mayor estabilidad de PINP que de CTX; poca estabilidad de la osteocalcina) o la posible interferencia por hemólisis o de sangre en orina (que afecta osteocalcina y NTX, respectivamente). No se han observado diferencias importantes entre que las medidas se realicen en suero o en plasma, si bien CTX es más estable en plasma que en suero, por lo que algunos autores sugieren utilizar plasma para estudios en que hay que congelar las muestras [15].

Finalmente, deberá considerarse que puede haber diferencias en relación al ensayo utilizado. Por ello, el laboratorio, ante cualquier cambio de ensayo deberá proceder a una comparación de las mediciones obtenidas entre ambos ensayos e informar al clínico de los resultados obtenidos. Obviamente, la disponibilidad de materiales de referencia internacionales conmutables y el desarrollo de procedimientos de medición comunes para los distintos biomarcadores óseos representaría un avance considerable en su aplicación clínica.

## References

[1] Brown JP, Don-Wauchope A, Douville P, Albert C, Vasikaran SD (2022). Current use of bone turnover markers in the management of osteoporosis. Clin Biochem.

[2] Schini M, Vilaca T, Gossiel F, Salam S, Eastell R (2023). Bone turnover markers: basic biology to clinical applications. Endocr Rev.

[3] Garnero P (2017). The utility of biomarkers in osteoporosis management. Mol Diagn Ther.

[4] Vasikaran S, Cooper C, Eastell R, Griesmacher A, Morris HA, Trenti T (2011). International Osteoporosis Foundation and International Federation of Clinical Chemistry and Laboratory Medicine position on bone marker standards in osteoporosis. Clin Chem Lab Med.

[5] Florez H, Hernández-Rodríguez J, Carrasco JL, Filella X, Prieto-González S, Monegal A (2022). Low serum osteocalcin levels are associated with diabetes mellitus in glucocorticoid treated patients. Osteoporos Int.

[6] Beck Jensen JE, Sørensen HA, Kollerup G, Jensen LB, Sørensen OH (1994). Biological variation of biochemical bone markers. Scand J Clin Lab Invest Suppl.

[7] Alvarez L, Ricós C, Peris P, Guañabens N, Monegal A, Pons F (2000). Components of biological variation of biochemical markers of bone turnover in Paget’s bone disease. Bone.

[8] European Federation of Clinical Chemistry and Laboratory Medicine (EFLM) Biological Variation Database. ..

[9] Cavalier E, Lukas P, Bottani M, Aarsand AK, Ceriotti F, Coşkun A (2020). European Federation of Clinical Chemistry and Laboratory Medicine Working Group on Biological Variation and IOF-IFCC Committee on Bone Metabolism. European Biological Variation Study (EuBIVAS): within- and between-subject biological variation estimates of β-isomerized C-terminal telopeptide of type I collagen (β-CTX), N-terminal propeptide of type I collagen (PINP), osteocalcin, intact fibroblast growth factor 23 and uncarboxylated-unphosphorylated matrix-Gla protein-a cooperation between the EFLM Working Group on Biological Variation and the International Osteoporosis Foundation-international Federation of Clinical Chemistry Committee on Bone Metabolism. Osteoporos Int.

[10] Diez-Perez A, Naylor KE, Abrahamsen B, Agnusdei D, Brandi ML, Cooper C (2017). Adherence Working Group of the International Osteoporosis Foundation and the European Calcified Tissue Society. International Osteoporosis Foundation and European Calcified Tissue Society Working Group. Recommendations for the screening of adherence to oral bisphosphonates. Osteoporos Int.

[11] Eastell R, Mallinak N, Weiss S, Ettinger M, Pettinger M, Cain D (2000). Biological variability of serum and urinary N-telopeptides of type I collagen in postmenopausal women. J Bone Miner Res.

[12] Smith ER, Cai MM, McMahon LP, Holt SG (2012). Biological variability of plasma intact and C-terminal FGF23 measurements. J Clin Endocrinol Metab.

[13] Cavalier E, Eastell R, Rye Jørgensen N, Makris K, Tournis S, Vasikaran S (2019). IFCC-IOF Joint Committee for Bone Metabolism (C-BM). A multicenter study to evaluate harmonization of assays for N-terminal propeptide of type I procollagen (PINP): a report from the IFCC-IOF Joint Committee for Bone Metabolism. Clin Chem Lab Med.

[14] Guañabens N, Filella X, Monegal A, Gómez-Vaquero C, Bonet M, Buquet D, LabOscat Study Group (2016). Reference intervals for bone turnover markers in Spanish premenopausal women. Clin Chem Lab Med.

[15] Cavalier E, Eastell R, Jørgensen NR, Makris K, Tournis S, Vasikaran S (2021). IFCC-IOF Committee for Bone Metabolism (C-BM). A multicenter study to evaluate harmonization of assays for C-terminal telopeptides of type I collagen (ß-CTX): a report from the IFCC-IOF Committee for Bone Metabolism (C-BM). Calcif Tissue Int.

[16] Bhattoa HP, Cavalier E, Eastell R, Heijboer AC, Jørgensen NR, Makris K, IFCC-IOF Committee for Bone Metabolism (2021). Analytical considerations and plans to standardize or harmonize assays for the reference bone turnover markers PINP and β-CTX in blood. Clin Chim Acta.

[17] Schafer AL, Vittinghoff E, Ramachandran R, Mahmoudi N, Bauer DC (2010). Laboratory reproducibility of biochemical markers of bone turnover in clinical practice. Osteoporos Int.

[18] Eastell R, Garnero P, Audebert C, Cahall DL (2012). Reference intervals of bone turnover markers in healthy premenopausal women: results from a cross-sectional European Study. Bone.

[19] Brown JP, Albert C, Nassar BA, Adachi JD, Cole D, Davison KS (2009). Bone turnover markers in the management of postmenopausal osteoporosis. Clin Biochem.

[20] Dincel AS, Jørgensen NR (2023). IOF-IFCC Joint Committee on Bone Metabolism (C-BM). New emerging biomarkers for bone disease: Sclerostin and Dickkopf-1 (DKK1). Calcif Tissue Int.

[21] Heijboer AC, Cavalier E (2023). The measurement and interpretation of fibroblast growth factor 23 (FGF23) concentrations. Calcif Tissue Int.

[22] Glover SJ, Gall M, Schoenborn-Kellenberger O, Wagener M, Garnero P, Boonen S (2009). Establishing a reference interval for bone turnover markers in 637 healthy, young, premenopausal women from the United Kingdom, France, Belgium, and the United States. J Bone Miner Res.

[23] Morris HA, Eastell R, Jorgensen NR, Cavalier E, Vasikaran S, Chubb SAP (2017). IFCC-IOF Working Group for Standardisation of Bone Marker Assays (WG-BMA). Clinical usefulness of bone turnover marker concentrations in osteoporosis. Clin Chim Acta.

[24] Glover SJ, Garnero P, Naylor K, Rogers A, Eastell R (2008). Establishing a reference range for bone turnover markers in young, healthy women. Bone.

[25] Adami S, Bianchi G, Brandi ML, Giannini S, Ortolani S, DiMunno O (2008). BONTURNO Study Group. Determinants of bone turnover markers in healthy premenopausal women. Calcif Tissue Int.

[26] Diemar SS, Dahl SS, West AS, Simonsen SA, Iversen HK, Jørgensen NR (2023). A systematic review of the circadian rhythm of bone markers in blood. Calcif Tissue Int.

[27] Gundberg CM, Markowitz ME, Mizruchi M, Rosen JF (1985). Osteocalcin in human serum: a circadian rhythm. J Clin Endocrinol Metab.

[28] Bollen AM, Martin MD, Leroux BG, Eyre DR (1995). Circadian variation in urinary excretion of bone collagen cross-links. J Bone Miner Res.

[29] Carpenter TO, Insogna KL, Zhang JH, Ellis B, Nieman S, Simpson C (2010). Circulating levels of soluble klotho and FGF23 in X-linked hypophosphatemia: circadian variance, effects of treatment, and relationship to parathyroid status. J Clin Endocrinol Metab.

[30] Herrmann M, Seibel MJ (2008). The amino- and carboxyterminal cross-linked telopeptides of collagen type I, NTX-I and CTX-I: a comparative review. Clin Chim Acta.

[31] Woitge HW, Knothe A, Witte K, Schmidt-Gayk H, Ziegler R, Lemmer B (2000). Circaannual rhythms and interactions of vitamin D metabolites, parathyroid hormone, and biochemical markers of skeletal homeostasis: a prospective study. J Bone Miner Res.

[32] Ashcherkin N, Patel AA, Algeciras-Schimnich A, Doshi KB (2023). Bone turnover markers to monitor oral bisphosphonate therapy. Cleve Clin J Med.

[33] Gass ML, Kagan R, Kohles JD, Martens MG (2008). Bone turnover marker profile in relation to the menstrual cycle of premenopausal healthy women. Menopause.

[34] Lombardi G, Lanteri P, Colombini A, Banfi G (2012). Blood biochemical markers of bone turnover: pre-analytical and technical aspects of sample collection and handling. Clin Chem Lab Med.

[35] Reynaga Montecinos B, Noemí Zeni S (2009). Marcadores bioquímicos del remodelamiento óseo. Utilidad clínica. Acta Bioquím Clín Latinoam.

[36] Clowes JA, Hannon RA, Yap TS, Hoyle NR, Blumsohn A, Eastell R (2002). Effect of feeding on bone turnover markers and its impact on biological variability of measurements. Bone.

[37] Vázquez-Sánchez S, Poveda J, Navarro-García JA, González-Lafuente L, Rodríguez-Sánchez E, Ruilope LM (2021). An overview of FGF-23 as a novel candidate biomarker of cardiovascular risk. Front Physiol.

[38] Moro Alvarez MJ (2001). Fármacos que afectan el metabolismo del hueso. Revista Española de Enfermedades Metabolicas Óseas.

[39] Eastell R, Szulc P (2017). Use of bone turnover markers in postmenopausal osteoporosis. Lancet Diabetes Endocrinol.

[40] Szulc P, Naylor K, Hoyle NR, Eastell R, Leary ET (2017). National Bone Health Alliance Bone Turnover Marker Project. Use of CTX-I and PINP as bone turnover markers: National Bone Health Alliance recommendations to standardize sample handling and patient preparation to reduce pre-analytical variability. Osteoporos Int.

